# The Duke and the Hospital

**Published:** 1891-07-18

**Authors:** 


### Passing Topics.

### THE DUKE AND THE HOSPITAL.

H.R.H. the Duke of Cambridge is not without a well-founded reputation for saying the right thing at the right time, and his observations at the opening of the new buildings of the London Hospital were distinguished by that good sense and thorough appreciation of the position which have, upon many occasions, marked the Duke's utterances in reference to hospital work. He, at least, comprehends some of the difficulties an unendowed hospital encounters, and is rightly prepared to give due weight to the unique conditions under which our voluntary hospitals render their services to the poor and the community.

To suppose the average critic, be he subscriber or, as is more often the case, non subscriber, capable of realising that a needy institution starts at a disadvantage, and that judgment to be just must be gentle, would be to misread all institutional history. A large number of people are always ready to condemn anything and everything which does not fit in with their own notions, and decide off-hand questions which would exercise considerably the minds of those who are more fully informed. Lately, we have had only too much evidence of a great and valuable organisation pursued by individual and collective rancour to be in any doubt as to how far a certain class of persons will be carried by their antipathies.

The acts of the London Hospital authorities furnish an ample answer to the charge often insinuated, if not actually alleged, that they are indifferent to the sanitary condition of their buildings. People are not ready to cover their indifference by an outlay of nine thousand pounds, and we can well believe that the Committee must have held very serious views indeed, to induce them to spend this large, but doubtless none too large, sum upon sanitation. It is obvious that a time comes in the history of every permanent organisation, when its arrangements and its methods need to be brought up to date. In the case of some of the older hospitals, it is certain that their sanitary condition is behind the times, but that is no reason for passing a harsh judgment upon their managers, and least of all is condemnation called for when the means are wanting to meet the expenditure which renovation entails.

"To condemn an establishment," said the Duke, "simply because a certain number of individuals have seen what they think may be better arranged seems most unjust and ungenerous. The only difficulty is that of getting the funds to carry out the work, but these are for the public to supply." Precisely: less criticism and more money is an old cry, but it still remains pertinent to the issue. The critics are more often than not a sorry lot when it comes to backing their opinions with their money. Earl Spencer has just borne testimony, as one of the Lords' Committee, to the marked ability with which most of our hospitals are conducted, but notwithstanding his Lordship's pleasant assertion we will make the critics a present of this admission—that if only they will supply the means to free the hospitals from debt and the many disabilities of perennial poverty, they shall see enough of reform and improvement to render their chosen vocation as superfluous then as now it is ungracious and presumptuous.

Agricola.

